# A zebrafish model of congenital nephrotic syndrome of the Finnish type

**DOI:** 10.3389/fcell.2022.976043

**Published:** 2022-09-14

**Authors:** Mi-Sun Lee, Sulochana Devi, John Cijiang He, Weibin Zhou

**Affiliations:** ^1^ Michigan Neuroscience Institute, University of Michigan, Ann Arbor, MI, United States; ^2^ Division of Nephrology, Department of Medicine, Icahn School of Medicine at Mount Sinai, New York, NY, United States

**Keywords:** nephrotic syndrome, nephrin, NPHS1, hypoalbuminemia, CRISPR/Cas9, zebrafish

## Abstract

Nephrotic syndrome (NS) is a disease characterized by proteinuria and subsequent hypoalbuminemia, hyperlipidemia and edema due to the defective renal glomerular filtration barrier (GFB). Mutations of NPHS1, encoding NEPHRIN, a podocyte protein essential for normal GFB, cause congenital nephrotic syndrome (CNS) of the Finnish type (CNF), which accounts for about 50% of CNS cases. We generated zebrafish nphs1 mutants by using CRISPR/Cas9. These mutants completely lack nephrin proteins in podocytes and develop progressive peri-orbital and whole-body edema after 5 days post fertilization. Ultra-structurally, loss of nephrin results in absence of slit-diaphragms and progressive foot process effacement in zebrafish pronephric glomeruli, similar to the pathological changes in human CNF patients. Interestingly, some nphs1 mutants are viable to adulthood despite ultra-structural defects in renal glomeruli. Using a reporter line Tg (l-fabp:VDBP-GFP) expressing GFP-tagged vitamin-D-binding protein in the blood plasma, we observed a reduction of intravascular GFP fluorescence in the nphs1 mutants, a hypoalbuminemia-like phenotype. In addition, we detected excretion of GFP by the nphs1 mutants, reminiscent of proteinuria. Therefore, we have demonstrated that the nphs1 mutant zebrafish recapitulate the human NS phenotypes and provide a novel and relevant animal model useful for screening therapeutical agents for this disease.

## Introduction

The kidney glomerulus is a selective blood filter, preserving cells and protein in the vascular space and filtering plasma. Nephrotic syndrome (NS) is caused by malfunction of kidney glomerular filters, resulting in proteinuria, consequent hypoalbuminemia, edema and hyperlipidemia. (Simic et al., 2013; Rheault and Gbadegesin, 2016). Primary causes of NS include minimal change disease, focal segmental glomerulosclerosis, membranous nephropathy, membranoproliferative glomerulonephritis and IgA nephropathy. Systemic diseases such as diabetes mellitus, amyloidosis and systemic lupus erythematosus also cause nephrotic syndrome (Johnstone and Holzman, 2006; Kawachi et al., 2009).

Proteinuria in NS is caused by abnormalities in glomerular podocytes, including podocyte foot-process retraction and slit diaphragm (SD) reorganization (Mizuno et al., 1999). Podocytes are highly specialized and polarized epithelial cells critical for renal glomerular filtration barrier that contribute to selectivity by series of interdigitating foot processes connected by the slit diaphragms. Podocytes are structurally and functionally conserved in vertebrates from zebrafish to humans (Reiser et al., 2000; Drummond., 2005). The SD is a specialized cell junction anchored to the actin cytoskeleton via a series of transmembrane proteins and is an essential element in filtration barrier function. Three kinds of SD-specific proteins Nephrin, Podocin, and Neph1 are localized at the SD with several tight junction-related proteins (Pavenstadt et al., 2003; Fukasawa et al., 2009). These SD-specific proteins are crucial for the formation and maintenance of the SD.

Genetic mutations in podocyte-related genes, such as NPHS1, NPHS2 and ACTN4, are known to be responsible for congenital NS (Doi et al., 2008). NEPHRIN, encoded by the NPHS1 gene, is crucial for the formation of the slit diaphragm, and mutations in NPHS1 cause congenital nephrotic syndrome of the Finnish type (CNF), an autosomal recessive steroid-resistant nephrotic syndrome (Holzman and Garg, 2009). The CNF progresses rapidly after birth and patients die within the first 2 years of life without treatment. The most classic clinic manifestation of CNF is heavy proteinuria, which begins *in utero*, along with hypoproteinemia and edema. NEPHRIN is an integral transmembrane protein of the immunoglobulin superfamily. In kidney, NEPHRIN is located exclusively at the filtration areas of the podocyte foot processes in the glomerulus (Kestila et al., 1998). Nephrin knockout (KO) mice develop edema and heavy non-selective proteinuria immediately after birth and lethality within 24 h. These mice exhibit the phenotypes consistent with the human CNF that include enlarged Bowman’s spaces, dilated tubules, and effacement of podocyte foot processes with the absence of SD. These results from the Nephrin KO mouse model confirm the essential role of NEPHRIN in slit diaphragm structure and function (Putaala et al., 2001).

Nephrin is functionally conserved in zebrafish (Ichimura et al., 2013; Fukuyo et al., 2014). In order to model the CNF using zebrafish, we generated zebrafish nphs1 mutants with CRISPR/Cas9-mediated genome editing and found that these mutants recapitulate the human CNF phenotypes and provides a novel and relevant disease model for *in vivo* functional studies of nephrin and for the screening of therapeutic agents targeting nephrin deficiency.

## Materials and methods

Zebrafish maintenance. Wild-type (AB strain) zebrafish were maintained in the zebrafish facility according to the University of Michigan Committee on Use and Care of Animals standards. All procedures were approved by the University of Michigan. Eggs were collected in petri-dishes and raised in incubators with a constant temperature of 28.5°C.

Generation of nphs1 mutant zebrafish. CRISPRscan (https://www.crisprscan.org/) and CHOPCHOP (https://chopchop.cbu.uib.no/) were used to design gRNAs targeting the exons of zebrafish nphs1 gene. The target sites were selected in early axons to potentially introduce early frame-shift mutations that maximize the loss of function of nephrin. A pair of forward: 5′- TAG​GGC​CTC​AGG​GGC​CGT​GCA​G-3′, reverse: 5′-AAA​CCT​GCA​CGG​CCC​CTG​AGG​C-3′ oligonucleotides were annealed and ligated into the BsaI-digested pDR274 vector (Addgene #42250). The gRNAs were *in vitro* synthesized using the Dra I-digested plasmids as templates using the MAXIscript T7 kit (Life Technologies). The Cas9 mRNA was *in vitro* transcribed using XbaI-digested pT3TS-Cas9 expression vector (Gift from Dr. Wenbiao Chen) and the mMESSAGE mMACHINE T7 kit (Life Technologies). The gRNAs and Cas9 mRNA were co-injected into one-cell stage zebrafish embryos (Gagnon et al., 2014). nphs1 heterozygote were crossed to the transgenic fish Tg (pod:gfp) or Tg (l-fabp:VDBP-EGFP) that were previously published (Zhou et al., 2010; Zhou and Hildebrandt, 2012) followed by genotyping and incrossing to generate nphs1-/- carrying the transgenes.

Genotyping and T7 endonuclease I (T7EI) mutation detection assays. Genomic DNA was extracted from single embryos or fin clips from adult fish. Targeted region was amplified from genomic DNA using specific primers. The PCR products were purified and T7 endonuclease I assays were performed as previously described (Sung et al., 2014). Sanger sequencing was performed using the FP primer. The genotyping primers: nphs1 FP (5′-TAA​GAC​GGA​GCC​GCG​TAA​TGT-3′); nphs1 RP (5′- CAC​CGC​ATG​TCA​TTC​CTC​CCT-3′).

Real-time qPCR. Total RNA was extracted from embryos with DirectZol-RNA extraction kit (Zymo Research) followed by first-strand cDNA synthesis (Invitrogen). Real-time PCR was performed using the SYBR Green Kit on the Applied Biosystems. ef1a is used for normalization for gene expression comparison. All reactions were performed in technical triplicates. The primers used for these experiments (nphs1 RT FP: 5′- GCC​TCG​ATG​TGC​TCT​TTC​CT-3′, nphs1 RT RP: 5′- TCG​TCT​GGG​TTT​GCA​GAG​AC-3′, nphs2 RT FP: 5′-CAG​TGT​GAG​GGA​ACG​GAT​AAA-3′, nphs2 RT RP: 5′- CAG​CTC​ACA​AAC​TCC​AAG​GTA-3′, ef1α RT FP: 5′- CTG​GAG​GCC​AGC​TCA​AAC​AT-3′, ef1α RT RP: 5′- ATC​AAG​AAG​AGT​AGT​ACC​GCT​AGC​ATT​AC-3′).

Whole-mount *in situ* hybridization. Zebrafish embryos were staged by morphological features and fixed in 4% PFA. Embryos were treated with 0.003% phenylthiourea (PTU) to inhibit pigment formation. Anti-sense RNA probes for nephrin and podocin were synthesized from cDNA templates using DIG-RNA labeling kit (Roche). Whole-mount *in situ* hybridization was performed as previously described (Thisse and Thisse, 2008).

JB-4 section for histology. Zebrafish embryos were fixed at 5 dpf in 4% paraformaldehyde, dehydrated with a graded ethanol series up to 100% and embedded in JB-4 Resin (Polytech) Cross sections (5 μm) were stained with hematoxylin and eosin (H&E) using standard protocols (Leica Microsystems).

Immunostaining and microscopy. For whole-mount immunofluorescence staining, 10 dpf zebrafish larvae were fixed overnight in 4% PFA and dehydrated with methanol. Samples were permeabilized in acetone for 7 min at -20°C and washed in water, followed by several washes in PBST. After blocking for 30 min in 2% sheep serum, samples were incubated with anti-Nephrin or anti-Podocin (Thermo Fisher #20384-1-AP) at 4 °C overnight and incubated with Alexa Fluor 568-conjugated secondary antibody (Invitrogen) during the following day. These Images were taken with Leica SP5 confocal microscope. Polyclonal anti-zebrafish nephrin antibody was raised in rabbits using its carboxy-terminal peptide RRDTDLP FELRGEL (1228–1241 amino acids) coupled to KLH. The antiserum was affinity-purified (Genscript, Nanjing, China).

Detection of proteinuria. nphs1+/-;Tg (l-fabp:VDBP-GFP) zebrafish were crossed and larvae carrying Tg (l-fabp:VDBP-GFP) were sorted by the edematous phenotype on 8 dpf. 30 nphs1-/- homozygotes or wild-type siblings were placed in 35 mm petri dish containing 1.5 ml of E3 embryo media supplemented with 0.5 ug/mL Kanamycin. The medium was collected 2 days later and processed with acetone to precipitate the proteins. The pellets were dissolved with 1x SDS sampling buffer (Boston Bioproducts) and subjected to Western blot to detect GFP following standard procedures. Primary antibody against EGFP (sc8334, Santa Cruz Biotechnology) was used at 1:4000 dilution.

Ultrastructural Analysis by Transmission electron microscopy (TEM). Zebrafish embryos were fixed in 0.1M sodium cacodylate buffer containing 4% paraformaldehyde and 2% glutaraldehyde (Electron Microscopy Sciences), post-fixed in 1% OsO4, and dehydrated through graded ethanol. TEM samples were embedded in Epoxy Resin. The ultrathin sections were stained with uranyl acetate and lead citrate. Images were taken under JEOL 1400-plus transmission electron microscope.

Statistical Analyses. The analytical statistics were generated in Prism (GraphPad Software). Throughout this report, data are presented as mean ± SD. n values are indicated by dots in histograms; every individual n value represents a different animal. Unpaired two-tailed *t*-test were used for statistical analyses.

## Results

### CRISPR/Cas9-induced zebrafish nphs1 mutants

**FIGURE 1:**
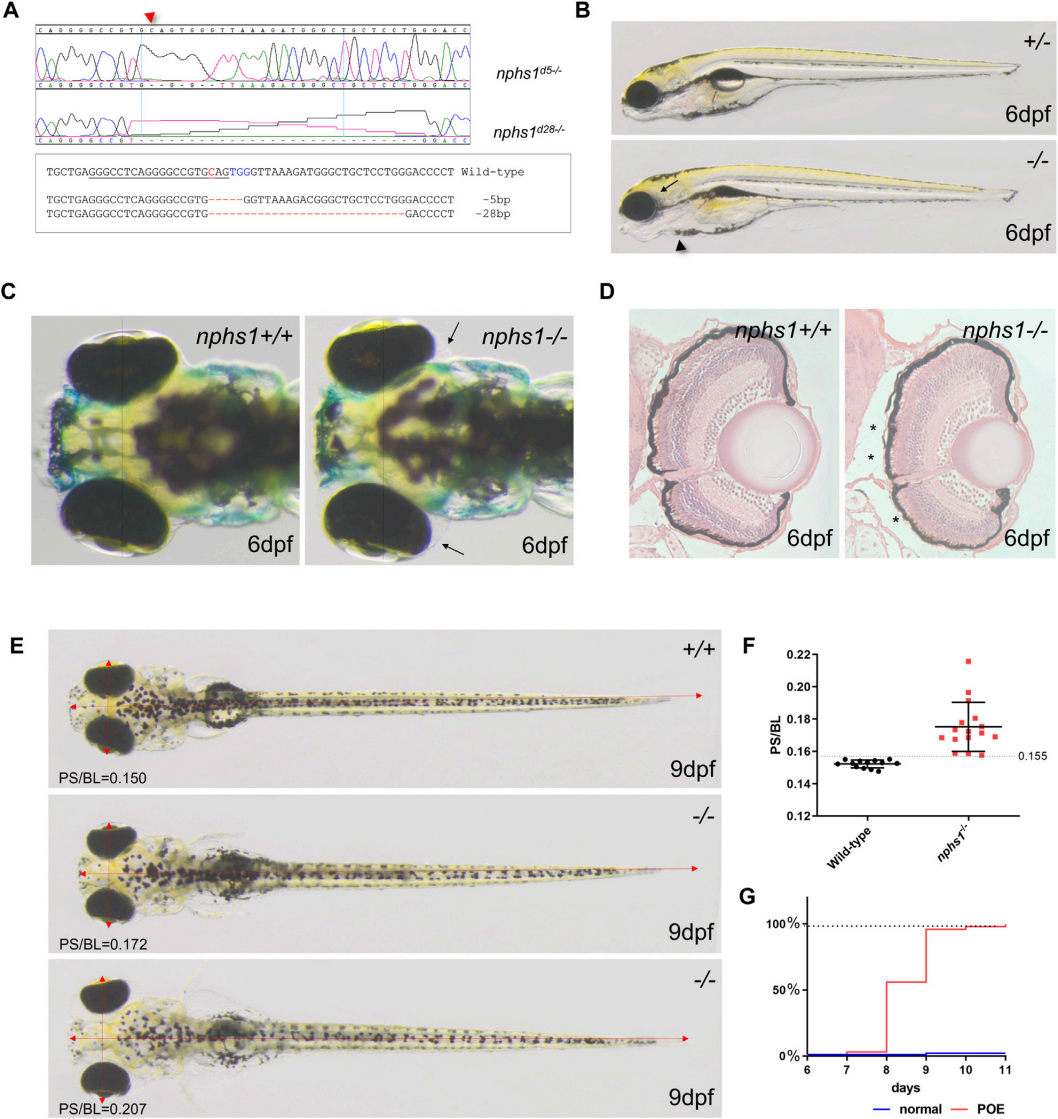
Generation of nphs1 mutant zebrafish by CRISPR/Cas9-mediated gene editing. **(A)** Sanger sequencing of the two different nphs1 mutant alleles. Sequence alignment of two nphs1 mutant alleles compared to the wild-type nphs1 gene sequence. PAMs are shown in blue color and sgRNA target sequence is underlined. The DNA break site by Cas9 is indicated red. **(B)** Morphological phenotype of nphs1 mutant at 6 dpf. The nphs1 mutant exhibited peri-orbital edema (arrow) and mild pericardial edema (arrowhead). **(C)** Dorsal view of the head region of zebrafish larvae. The nphs1 mutants exhibit peri-orbital edema (arrows) compared to wild-type siblings at 6 dpf. **(D)** Cross sections of periorbital region in wild-type and nphs1 mutants at 6 dpf. H&E staining shows the severity of peri-orbital edema (asterisks). **(E)** nphs1-/- develops severe periorbital and whole-body edema at 9 dpf compared to wild-type siblings. Red arrows indicate the pupil spacing distance (PS) and the body length (BL). **(F)** Scatter plot showing measurement of PS/BL in nphs1 mutants at 9 dpf. Each data point represents an individual larva and lines indicate the mean values. The error bars show the standard error of the mean (SEM). **(G)** Progression of the peri-orbital edema in nphs1 mutants (red line) and wild-type controls at 6–11 dpf.

The zebrafish nephrin protein has a high level of similarity in the conserved domain to its human and mouse orthologue (Kramer-Zucker et al., 2005) (Supplementary Figure S1A). To model the nphs1 deficiency in zebrafish, we utilized the CRISPR/Cas9 technology (Mali et al., 2013; Chang et al., 2013; Hwang et al., 2013; Jao et al., 2013; Sung et al., 2014) to generate genetic mutants. We screened and designed gRNAs to target exon 2 of nphs1 gene encoding part of the immunoglobulin like (IG_like) domain (Supplementary Figure S1B). The effectiveness of CRISPR-induced mutagenesis in F0 embryos was demonstrated by T7 endonuclease I (T7EI) assay (Supplementary Figure S1C). A small fraction of the larvae injected with the gRNA/Cas9 exhibited severe edema at 7 days post fertilization (dpf) (Supplementary Figure S1D), resembling the nphs1 morphants (Kramer-Zucker et al., 2005), presumably due to the bi-allelic deletions induced by CRISPR/Cas9. We screened for the germline transmission of the nphs1 mutations and established two mutant lines of nphs1 with 5 bp and 28 bp deletions, respectively (Figure 1A). These two mutant alleles were predicted to result in a premature stop codon in the coding sequence due to reading frame shift, leading to a truncated nephrin protein lacking the immunoglobulin like (IG_like) domain (Supplementary Figure S2A–B).

### The nphs1 mutants exhibit late on-set edematous phenotypes

We characterized nphs1 mutant phenotypes with both mutant alleles to minimize the possibility of erroneous off-target editing by CRISPR. Neither nphs1-/- mutants produced any obvious morphological abnormality until after 5 dpf, when peri-orbital and pericardial edema were visible (Figures 1B,C). As nphs1-/- exhibited severe peri-orbital edema phenotypes, we analyzed the morphology of the retina of the nphs1 mutants with histological staining and found no obvious retinal defect (Figure 1D). To characterize the phenotypic progression, we adopted the pupil-spacing (PS) distance and the body length (BL) ratio as a metric of the severity of peri-orbital edema and established that PS/BL in wild-type larvae is no greater than 0.155 (n = 13) (*p* < 0.001, *t*-test) at 9 dpf (Figures 1E,F). At 8 dpf, a little more than 50% of nphs1-/- developed periorbital edema, and by 10 dpf, more than 95% of the mutants had severe peri-orbital edema (Figure 1G). Thus, nphs1-/- displayed edematous phenotypes, resembling other zebrafish models of podocyte defects (Wan et al., 2016; Jobst-Schwan et al., 2019) and reminiscent of edema seen in pediatric patients with NS. This progressed into severe whole-body edema and lethality occurred around 2 weeks of age (Supplementary Figure S2C), similar to Nephrin KO mice that are lethal shortly after birth. However, unexpectedly, very few nphs1-/- (3.23%, n = 2 out of 62 fishes) (Supplementary Figure S2D), showed no edematous phenotype and were viable to adulthood.

### The nphs1 mutants lack nephrin protein expression in podocytes

**FIGURE 2:**
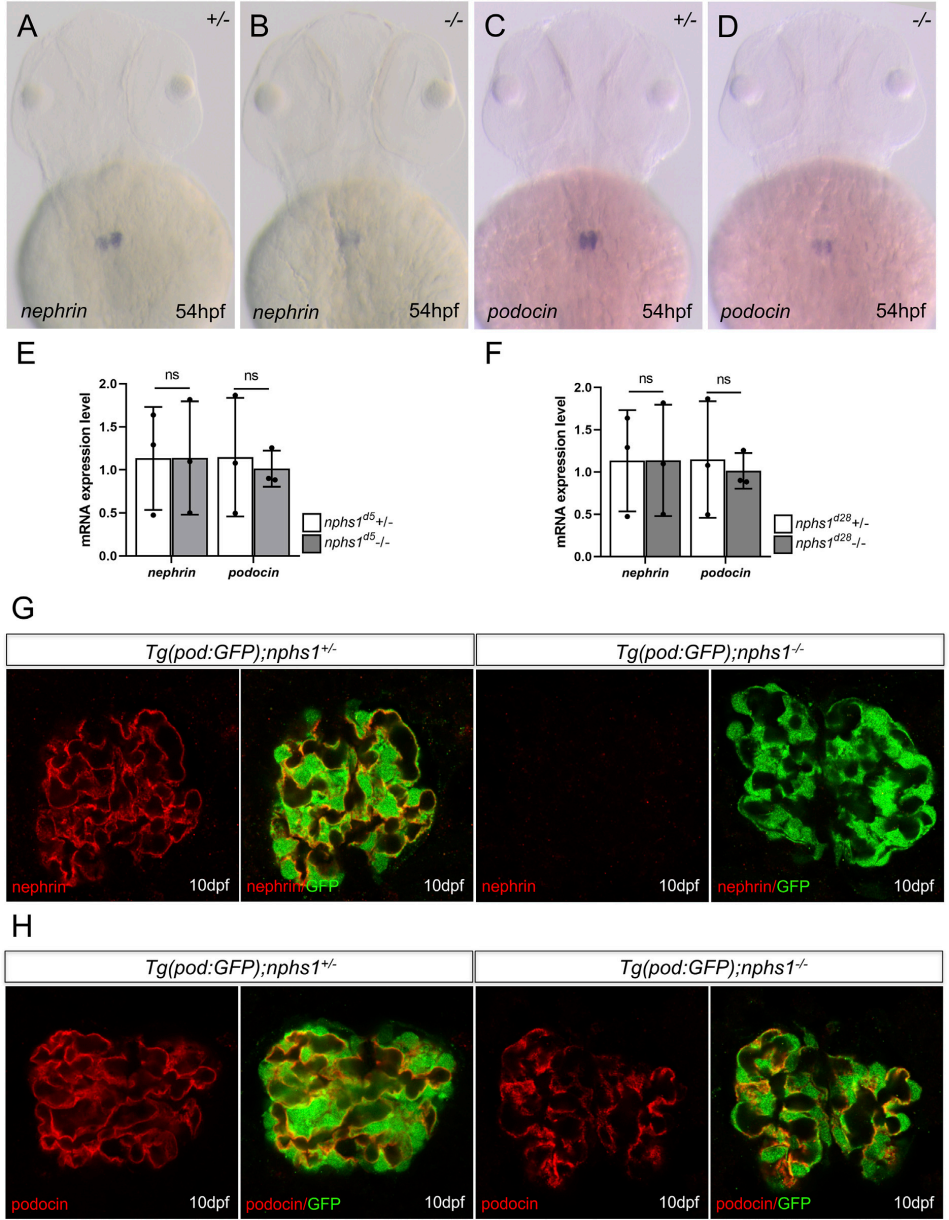
The expression analyses of nephrin and podocin in nphs1 mutants. Whole-mount *in situ* hybridization for nephrin **(A–B)** and podocin **(C–D)** at 54 hpf **(E–F)** Quantitative PCR analyses of mRNA levels of nphs1 and nphs2 show no statistically significant difference between nphs1 heterozygotes (+/-) and nphs1 homozygous mutants (-/-) at 7 dpf for both 5bp deletion (d5) and 28bp deletion (d28) mutant alleles. The expression level is normalized to that of eflα. The error bars show the standard error of the mean (SEM). **(G–H)** Confocal images of Immunofluorescence staining for nephrin (G, red) and podocin (H, red) at 10 dpf. Podocytes are marked by GFP expression (green) by the Tg (pod:GFP) transgene. No nephrin expression is detected in Tg (pod:GFP); nphs1-/- at 10 dpf, while podocin is present with a grainy pattern.

Zebrafish nphs1 and nphs2 are specifically expressed in pronephric podocytes and required for the development of glomerular filtration barriers. Both nephrin and podocin localize at the slit diaphragms between the podocyte foot processes and play essential roles in the formation and maintenance of the slit diaphragms (Fukuyo et al., 2014). We first examined nphs1 and nphs2 expression by whole-mount *in situ* hybridization and found that both genes were expressed in the pronephric podocytes in nphs1 homozygotes as well as in the siblings at 54 h post fertilization (hpf) (Figures 2A–D). Similarly, no significant difference between nphs1+/− and nphs1-/- in the transcript levels of podocin and nephrin for both nphs1 mutant alleles (Figures 2E,F). These results suggest that these two nphs1 mutant alleles do not affect mRNA stability or transcript levels of nphs1 and nphs2. Next, we investigated the protein expression of nephrin and podocin within the pronephric glomerulus by immunofluorescence staining and confocal microscopy. Nephrin was previously reported to localize predominantly at the periphery the podocytes within the pronephros, similar to its localization in the mammalian metanephros (Kramer-Zucker et al., 2005; O’Brien et al., 2011; Ichimura et al., 2013; Fukuyo et al., 2014). We observed that nephrin and podocin localized to major processes and foot processes of podocytes in pronephric glomeruli in nphs1+/− at 10 dpf (Figures 2G,H). However, we could not detect any signal above background with an antibody against zebrafish nephrin in nphs1-/-, while podocin expression in nphs1-/- displayed a grainy pattern compared to the continuous pattern in nphs1+/− at 10 dpf (Figure 2H). This suggests the disrupted organization of slit diaphragms in the absence of nephrin. Thus, both nphs1 mutant alleles completely abolished the protein expression of nephrin without affecting its mRNA level.

### Mutation in nphs1 induce foot process effacement in zebrafish

**FIGURE 3:**
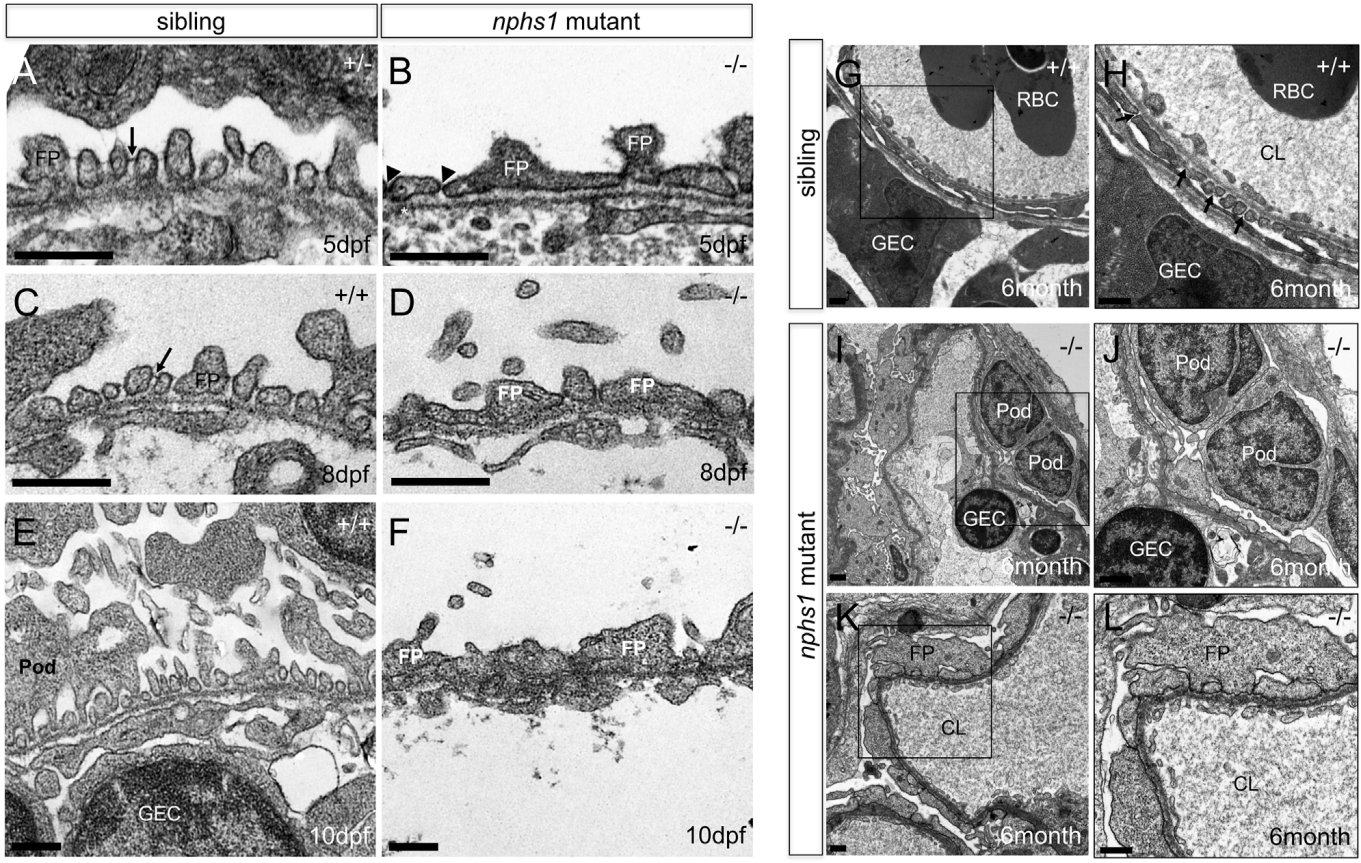
Lack of nephrin results in absence of slit-diaphragms and foot process effacement. **(A,C,E)** Transmission Electron Micrographs (TEM) of the podocytes foot processes in wild type zebrafish at 5, 8, 10 dpf. Presence of slit diaphragms are marked with arrows. **(B,D,F)** TEM of the podocyte foot processes in nphs1-/- at 5, 8, 10 dpf showing widened and irregular foot processes effacement with lack of detectable slit diaphragms (arrowheads) (Scale bar, 400 nm). **(G–H)** TEM of the mesonephric podocytes foot processes in 6-month-old adult wild-type siblings. Presence of slit diaphragms are marked with arrows. **(I–L)** TEM of the mesonephric podocytes foot processes in viable nphs1-/- adults. The foot processes are effaced and widened with absence of slit diaphragm throughout capillary wall in 6-month-old adult nphs1 mutants. **(H,J,L)** Higher-magnification images show podocyte foot-process effacement in nphs1-/- adults. Pod: podocyte; FP: foot process, GEC: glomerular endothelial cells; CL: glomerular capillary lumen; RBC: red blood cell.

Using Tg (pod:GFP), a transgenic line in which podocytes are labeled with GFP (Zhou et al., 2010), we did not observe with confocal microscopy obvious podocyte loss in nphs1-/- at 10 dpf when those mutants have already developed severe periorbital and whole-body edema (Figures 2G,H). We next utilized transmission electron microscopy (TEM) to determine if the nphs1 mutations caused any structural defects of podocytes. In wild-type siblings, normal podocyte foot processes and slit diaphragms were clearly visible at as early as 5 dpf (Figure 3A). However, the nphs1 mutants exhibited irregularly broadened foot processes and absence of slit diaphragm (Figure 3B) at 5 dpf when edema was barely noticeable. At 8 dpf and 10 dpf, regular foot processes with slit diaphragms covered surface of the glomerular basement membrane in wild-type sibling (Figures 3C,E), but nphs1 mutants had irregularly shaped foot processes and the slit diaphragms were completely missing between abnormal foot processes (Figures 3D,F). These ultrastructural defects in podocytes are consistent with Nephrin KO mice and human patients of CNF and indicate that nphs1 is required for the formation of normal foot processes and slit diaphragms. We also examined the mesonephric podocytes in the viable adult nphs1 mutants with TEM. Compared to wild-type adult fish that have normal foot processes and slit diaphragms in the mesonephric glomeruli (Figures 3G,H), nphs1-/- adults clearly had foot processes effacement and lacked slit diaphragms (Figures 3I–L). However, interestingly, the protein cast within the capillary lumen appeared comparable between the wild-type siblings and the adult nphs1-/- (Figures 3K,L). This suggests that the adult nphs1-/- mutants did not have significant loss of serum proteins even though the abnormal ultrastructure of podocytes. It is also consistent with the lack of edematous phenotypes in these viable mutants. We did not observe loss of podocytes or denuded capillaries in the viable adult nphs1 mutants, and thus the pathological feature of the podocytes is more similar to that of minimal change disease rather than focal segmental glomerular sclerosis.

**FIGURE 4:**
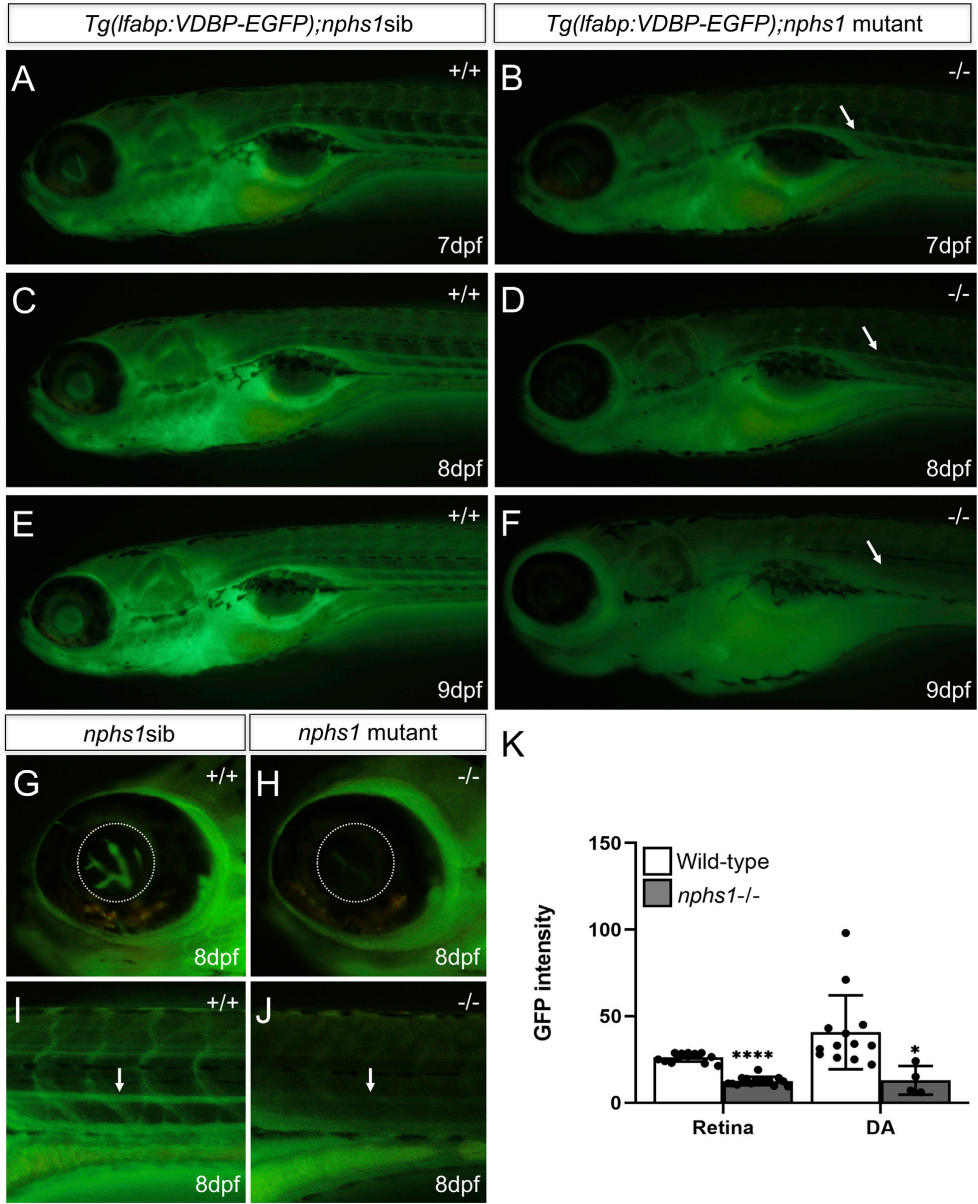
Measurement of hypoalbuminemia-like phenotype in nphs1-/- zebrafish. **(A,C,E)** The GFP fluorescence in wild-type siblings with Tg (l-fabp:VDBP-EGFP) at 7-9 dpf. **(B,D,F)** The GFP fluorescence in nphs1-/- mutants with Tg (l-fabp:VDBP-EGFP) at 7-9 dpf. **(G–J)** The GFP fluorescence in retina vessels and dorsal aorta of wild type fish **(G,I)** and of nphs1-/- mutants **(H,J)** at 8 dpf. **(K)** The quantitative measurements of the GFP fluorescence intensity in the retinal vessels **(G,H)** and the dorsal aorta **(I–J)** (arrow) using ImageJ. The error bars show the standard error of the mean (SEM) (*p ≤ 0. 1, ****p ≤ 0. 0001) (Student’s t-test).

### Deficiency of nphs1 results in a phenotype mimicking hypoalbuminemia and proteinuria

In NS, proteinuria due to defective GFB leads to non-selective loss of serum proteins and hence hypoalbuminemia. Albumin is one of the major serum proteins that are present in proteinuria in mammals. However, there is no identifiable albumin orthlog in zebrafish genome, while the vitamin-D-binding protein (VDBP) contains two albumin domains and is also one of the major serum proteins closely related to albumin. To evaluate the GFB function in nphs1-/- zebrafish, we produced nphs1-/- with Tg (l-fabp:VDBP-GFP), in which GFP tagged VDBP is produced in the liver and secreted into blood (Zhou and Hildebrandt, 2012). We found the wild type Tg (l-fabp:VDBP-GFP) larvae showed an increase of overall fluorescence throughout the fish during 7-9 dpf (Figures 4A,C,E), but the nphs1-/- mutants had decreased GFP fluorescence with the development of edematous phenotypes (Figures 4B,D,F). In order to establish a new assay applicable for high-throughput screening, we used this reporter line and fluorescence microscopy to measure the intravascular fluorescence as a surrogate for the abundance of serum proteins. (Figures 4G–J). We found that the GFP fluorescence was significantly reduced in nphs1-/- in both the retinal vessel plexus of eye and the dorsal aorta at 8 dpf compared to the wild-type siblings (Figure 4K), indicating that nphs1 mutations led to loss of VDBP-GFP due to leaky GFB, a phenotype resembling hypoalbuminemia seen in NS patients. We also detected by Western blot the GFP protein (presumably the degradation product of VDBP-GFP fusion protein) in zebrafish culture media for the nphs1 mutants but not for their wild-type siblings (Supplementary Figure S3). This confirmed the proteinuria that resulted from defective GFB in the mutant fish.

## Discussion

In this study, we generated two zebrafish mutant alleles of nphs1 by CRISPR genome editing. Both nphs1 mutants completely lost nephrin protein expression in pronephric podocytes, suggesting complete loss of function of nphs1, but compared to previously reported morpholino-mediated knockdown of nphs1 in zebrafish that causes severe pericardial edema and body curvature at 3 dpf (Fukuyo et al., 2014), our nphs1 mutants appear to have delayed edema and lethality and absence of body curvature. They did not develop any observable edema until after 5 dpf and the pericardial edema was rather mild. A possible reason is that off-target effects of MOs and MO-induced of p53 may have exacerbated the podocyte defects in nphs1 morphants (Gerety and Wilkinson, 2011). Although Genetic compensation can be induced by non-sense-mutation-mediated mRNA degradation in genetic mutants but not in morphants (Rossi et al., 2015; El-Brolosy and Stainier, 2017), we have not detected in nphs1-/- the decrease of the nphs1 mRNA level, which diminishes this possibility for our nphs1 mutants. Nephrin knockout mice are lethal shortly after birth due to massive proteinuria. In contrast, we found a small percentage of nphs1 mutants were viable to adulthood without obvious edema or podocyte loss albeit maintaining the foot process defects in mesonephros. It is noteworthy that Nephrin is absent in the slit diaphragm in birds (Casotti and Braun, 1996; Volker et al., 2012) in order to have larger SD as compared with mammalian glomeruli and for proper excretion of nitrogen in the form of uric acid. Therefore, whether nphs1 deficiency can be compensated by other podocyte proteins in zebrafish or the defective glomerular filtration can be compensated by other physiological mechanism(s) in zebrafish requires further investigations. This may provide new insights for the therapy of NS.

It is technically challenging to assess the GFB function directly in zebrafish. Nevertheless, we were able to detect the excessive GFP protein in the concentrated zebrafish culture media for nphs1-/- carrying the VDBP-GFP transgene as a serum protein tracer by Western blot with an antibody against GFP. This demonstrated the proteinuria in nphs1 mutants. Moreover, in order to develop an assay for large-scale screening of therapeutic agents to treat the GFB defects, we showed the feasibility to use fluorescence-based measurement of intravascular protein abundance to examine the hypoalbuminemia-like phenotype due to the defective GFB in the nphs1 mutants. In addition, given the convenience and efficiency of generating transgenic zebrafish, our new zebrafish model is potentially a useful platform to perform functional studies of disease-causing alleles of NPHS1 identified in human patients of CNF and other glomerular diseases.

In summary, our zebrafish model of CNF recapitulated the podocyte defects, such as lack of slit diaphragm and foot process effacement, in human CNF patients with NPHS1 mutations and Nephrin KO mice. This demonstrates that nphs1 gene function is conserved in zebrafish and these mutants provide a new and relevant disease model for future *in vivo* studies.

## Data Availability

The raw data supporting the conclusion of this article will be made available by the authors, without undue reservation.
