# Supplementary figures and images for: A zebrafish model of congenital nephrotic syndrome of the Finnish type
Source: Front Cell Dev Biol. 2022 Sep 14;10:976043. doi: 10.3389/fcell.2022.976043 (PMC9515793; doi:10.3389/fcell.2022.976043)

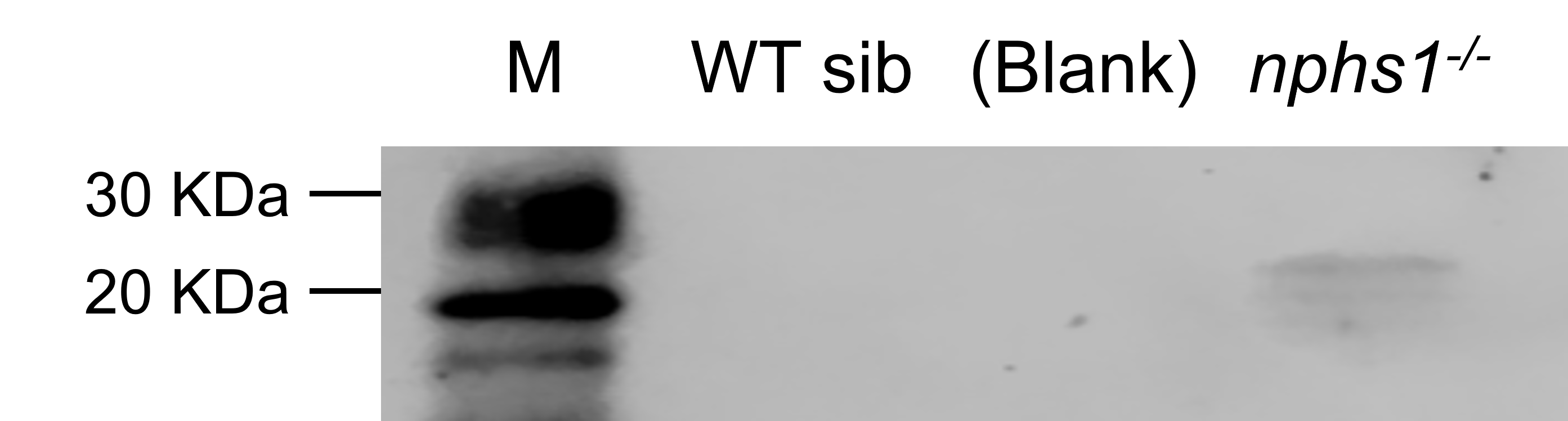

Supplement: Supplementary file 1 [file Image3.TIF]

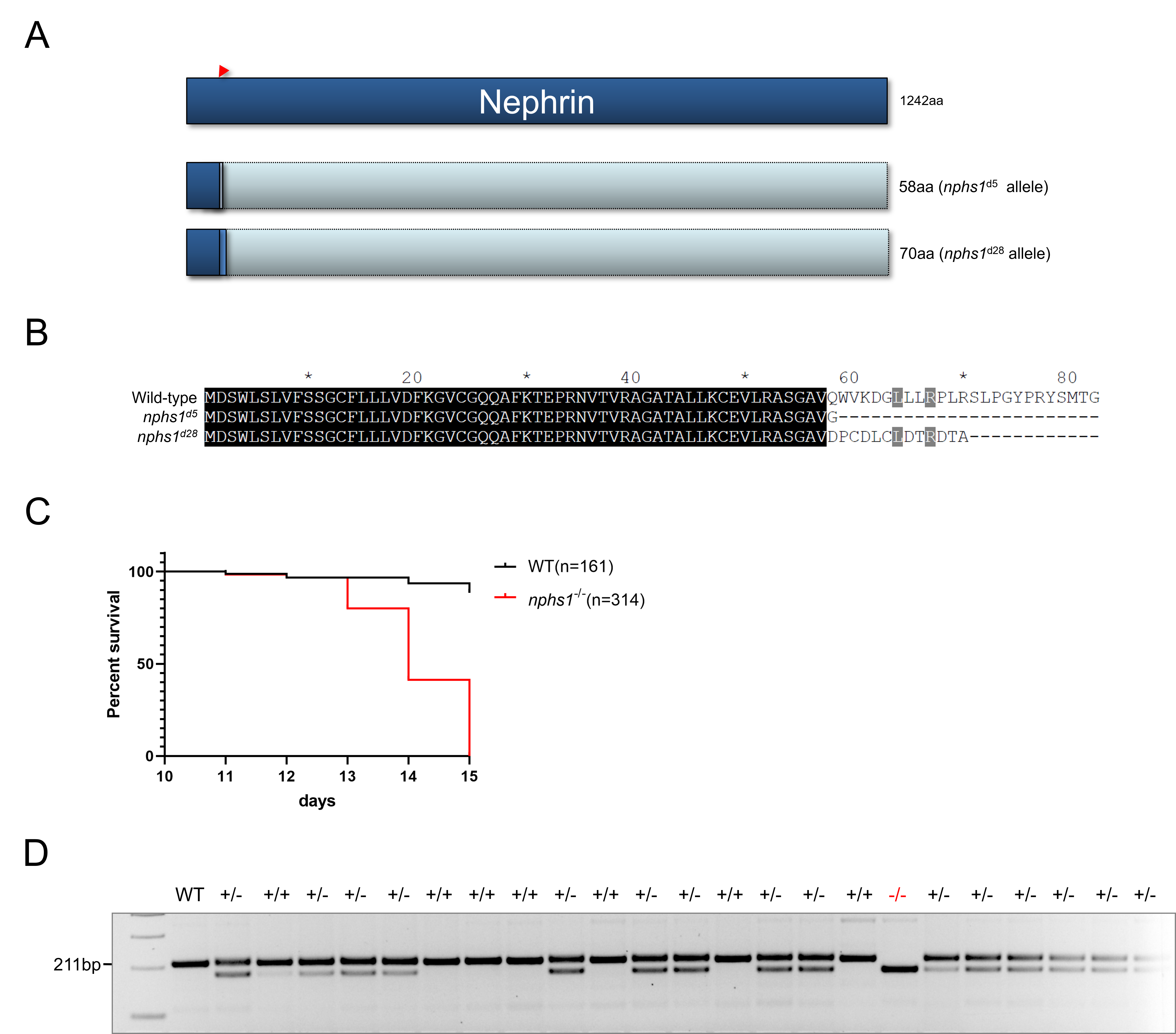

Supplement: Supplementary file 2 [file Image2.TIF]

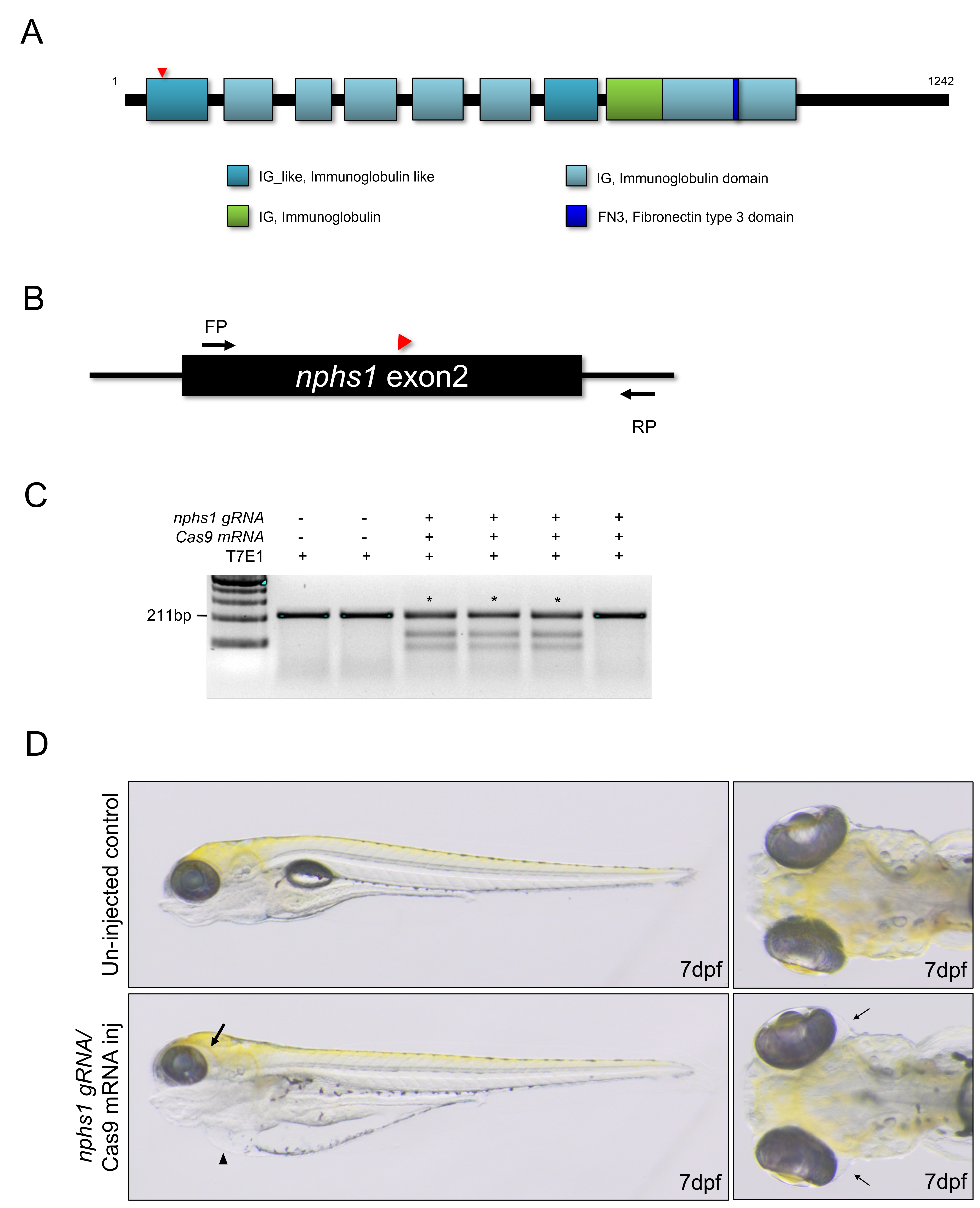

Supplement: Supplementary file 3 [file Image1.TIF]
